# Hydroxyurea reduces leukocyte interactions with the vessel wall in a haemolytic mouse model via a possible NO/cGMP-mediated effect

**DOI:** 10.1186/2050-6511-14-S1-P2

**Published:** 2013-08-29

**Authors:** Camila B Almeida, Claudio C Werneck, Cristina C Vicente, Fábio T Costa, Fernando F Costa, Nicola Conran

**Affiliations:** 1 Hematology and Hemotherapy Center, UNICAMP, Campinas, São Paulo 13083-878, Brazil; 2 Department of Biochemistry, Institute of Biology, UNICAMP, Campinas, São Paulo 13083-970, Brazil; 3 Department of Genetic, Evolution and Bioagents, Institute of Biology, UNICAMP, Campinas, São Paulo 13083-970, Brazil

## Background

Haemolysis occurs in a number of haematologic and non-haematologic diseases, including sickle cell disease, malaria and sepsis. Elevated extracellular haemoglobin (Hb) can trigger specific events that are associated with adverse clinical outcomes. For example, in sickle cell disease, cell-free haemoglobin has been proposed to reduce nitric oxide (NO) bioavailability and induce vascular oxidative stress and, possibly, inflammation [1]. Hydroxyurea, a drug used commonly as a therapy for sickle cell disease, may exert some of its effects by acting as a NO donor, *in vivo *[2]. This study aimed to compare the effects of haemolytic and inflammatory stimuli on blood vessel leukocyte recruitment *in vivo*. The effects of the acute administration of hydroxyurea (HU) or an NO donor on these alterations were also investigated.

## Methods

**Figure 1:**
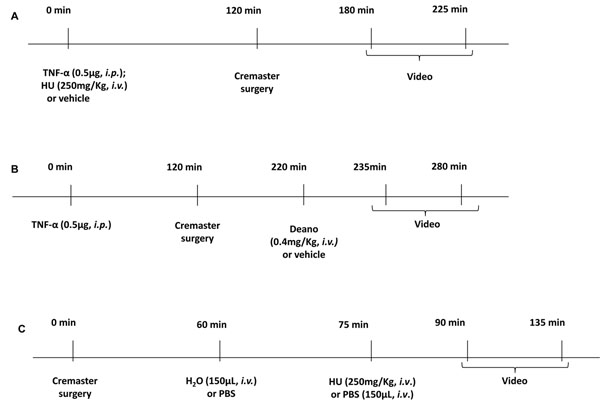
Protocols used in IVM

Inflammatory or hemolytic processes were induced in C57BL/6 mice (2-4 months old) and cremaster muscles of mice were prepared for intravital microscopy (IVM), according to protocols described in figure 1. Plasma-free Hb was measured with Drabkin's solution.

## Results

C57BL/6 mice that received water *i.v.* (H_2_O mice) presented marked vascular haemolysis after 15 min; levels of plasma Hb were more than doubled in H_2_O mice, compared to mice that receiving PBS *i.v.* (2.57±0.43g/dL, 1.08±0.08g/dL Hb, respectively, p<0.01, n=3-5), and resembled those of SCD mice (data not shown). In contrast, C57BL/6 mice (n=3) that received an inflammatory stimulus (TNF-α) showed no alteration in plasma free Hb levels (1.29±0.43 g/dL, 1.23±0.09 g/dL; PBS and TNF-α, respectively). IVM demonstrated that both the inflammatory and haemolytic stimuli induced leukocyte adhesion to vessel walls (3.57±0.84; 11.07±0.92; 7.25±1.20 [100µm^-1^] for PBS, TNF-α and H_2_O, respectively, p<0.05, N=15-30 venules), as well as leukocyte extravasation (1.07±0.17; 4.02±0.39; 2.97±0.49 [per 100 µm x 50 µm] for PBS, TNF-α and H_2_O, respectively, p<0.05, N=15-30 venules). Surprisingly, when HU was given simultaneously with TNF-α or following H_2_O, this drug was able to prevent leukocyte recruitment in both models, reducing both leukocyte adhesion (7.40±0.47; 2.79±0.34 [100 µm^-1^] for TNF-α+PBS and TNF-α+HU, respectively, p<0.0001, n=30-60 venules; and 7.84±0.73; 2.41±0.37 for H_2_O+PBS and H_2_O+HU, p<0.0001, n=20-30 venules) and leukocyte extravasation (3.88±0.26; 1.22±0.18 for TNF-α+PBS and TNF-α+HU, p<0.0001, n=30-60 venules and 2.98±0.32; 1.94±0.21 for H_2_O+PBS and H_2_O+HU, p<0.0001, n=20-30 venules) and increased leukocyte rolling (18.11±1.58; 25.48±5.01 for TNF-α+PBS and TNF-α+HU, respectively, p<0.05, n=30-60 venules and 11.56±1.47; 32.47±3.54 for H_2_O+PBS and H_2_O+HU, p<0.0001, n=20-30 venules). Additionally, DEANO was also able to reverse the inflammatory process installed by TNF-α, reducing leukocyte adhesion (10.24±1.49; 11.45±1.40 for TNF-α before and after vehicle control, n=21-22 venules; 7.63±0.63; 3.76±0.41 for TNF-α, before and after DEANO, p<0.01; n=29-35 venules) and extravasation (1.95±0.29; 3.23±0.44 before and after vehicle control, p<0.05, n=21-22 venules; 2.49±0.25; 2.24±0.24 before and after DEANO, n=29-35 venules).

## Conclusion

The induction of intravascular haemolysis in mice may rapidly induce alterations in leukocyte recruitment in vessels, similar to those observed following a substantial inflammatory stimulus. Acute administration of HU was found to reverse the effects of both inflammatory and haemolytic stimuli, in a manner similar to an established NO donor (DEANO). Results indicate that HU appears to have immediate beneficial effects in blood vessels. Data support growing evidence suggesting that one of the principal mechanisms by which HU exerts its effects *in vivo* is by a nitric oxide donor/cGMP-stimulating effect; moreover, targeting the NO/cGMP may be an important approach for therapies for haemolytic, as well as for inflammatory, diseases. Further experiments are underway to confirm that HU acts via NO/cGMP in the haemolytic model.
